# Functional Imaging of Numerical Processing in Adults and 4-y-Old Children

**DOI:** 10.1371/journal.pbio.0040125

**Published:** 2006-04-11

**Authors:** Jessica F Cantlon, Elizabeth M Brannon, Elizabeth J Carter, Kevin A Pelphrey

**Affiliations:** **1**Department of Psychological and Brain Sciences, Duke University, Durham, North Carolina, United States of America; **2**Center for Cognitive Neuroscience, Duke University, Durham, North Carolina, United States of America; **3**Brain Imaging and Analysis Center, Duke University, Durham, North Carolina, United States of America; Service Hospitalier Frederic JoliotFrance

## Abstract

Adult humans, infants, pre-school children, and non-human animals appear to share a system of approximate numerical processing for non-symbolic stimuli such as arrays of dots or sequences of tones. Behavioral studies of adult humans implicate a link between these non-symbolic numerical abilities and symbolic numerical processing (e.g., similar distance effects in accuracy and reaction-time for arrays of dots and Arabic numerals). However, neuroimaging studies have remained inconclusive on the neural basis of this link. The intraparietal sulcus (IPS) is known to respond selectively to symbolic numerical stimuli such as Arabic numerals. Recent studies, however, have arrived at conflicting conclusions regarding the role of the IPS in processing non-symbolic, numerosity arrays in adulthood, and very little is known about the brain basis of numerical processing early in development. Addressing the question of whether there is an early-developing neural basis for abstract numerical processing is essential for understanding the cognitive origins of our uniquely human capacity for math and science. Using functional magnetic resonance imaging (fMRI) at 4-Tesla and an event-related fMRI adaptation paradigm, we found that adults showed a greater IPS response to visual arrays that deviated from standard stimuli in their number of elements, than to stimuli that deviated in local element shape. These results support previous claims that there is a neurophysiological link between non-symbolic and symbolic numerical processing in adulthood. In parallel, we tested 4-y-old children with the same fMRI adaptation paradigm as adults to determine whether the neural locus of non-symbolic numerical activity in adults shows continuity in function over development. We found that the IPS responded to numerical deviants similarly in 4-y-old children and adults. To our knowledge, this is the first evidence that the neural locus of adult numerical cognition takes form early in development, prior to sophisticated symbolic numerical experience. More broadly, this is also, to our knowledge, the first cognitive fMRI study to test healthy children as young as 4 y, providing new insights into the neurophysiology of human cognitive development.

## Abstract 2

This functional imaging study provides evidence for a neurobiological link between early non-symbolic numerical abilities of 4 year-old children and the more symbolic numerical processing of adults.

## Introduction

Intuitively, language influences the way we think about number. However, substantial evidence indicates that preverbal children and human adults, as well as other animals, share a fundamental mechanism for representing approximate numerical values that is independent of language [ 1– 10]. Further, humans appear to possess a common psychological currency for representing numerical value regardless of whether the value is communicated symbolically via Arabic numerals and number words or non-symbolically through the number of visual objects in a set or the number of tones in an auditory sequence [ 3, 11– 14]. These and other findings have led researchers to predict that approximate numerical information, whether symbolic or non-symbolic, is processed by a common neural substrate [ 15– 17]. Neuroimaging and lesion studies of adult humans have demonstrated that the intraparietal sulcus (IPS) plays a central role in processing symbolic numerical information [ 18– 20]. For example, people with damage to parietal cortex have difficulty identifying which of two Arabic numerals is larger, or computing which numeral falls between two others [ 19], and damage specifically to the IPS has been reported to cause acalculia, a severe mathematical deficit [ 21]. Several neuroimaging studies have reported increased activity in the IPS when adult participants perform approximate arithmetic operations on Arabic numerals relative to control tasks [ 21– 25]. The IPS also responds more strongly when adult participants engage in a number word or Arabic numeral detection task than a color detection task [ 26]. The IPS further shows the effects of repetition suppression when numerals are primed at subconscious thresholds and perceptually masked [ 27]. By adulthood, the IPS is clearly active during symbolic numerical operations. However, a critical and controversial question is whether the IPS is also important for processing non-symbolic numerical magnitude and therefore processes number irrespective of notation [ 28, 29, 30]. While behavioral studies of adults implicate a link between approximate symbolic and non-symbolic numerical processing, neuroimaging studies have yielded conflicting results on a link between these two types of numerical processing.

From an early age, young children are sensitive to the numerical attributes of stimuli, and their non-symbolic numerical abilities exhibit important continuities with those of adults. Like adult humans, when young children compare the numerical values of sets of objects (e.g., arrays of dots), their performance is dependent on the ratio between the values rather than the absolute values (for adults see [ 3, 11, 12]; for children see [ 14, 31– 33]). For example, both adults and young children are faster and more accurate at comparing numerical values when the ratio between them is small (e.g., 6 versus 9 = 2/3 ratio) than when it is large (e.g., 4 versus 5 = 4/5 ratio). This capacity for approximate non-symbolic numerical estimation shows the same signature of ratio-dependent discrimination in human infancy [ 4, 34– 39]. Evidence that number discrimination is ratio-dependent throughout development and in adulthood suggests that the physiological basis for numerical processing is developmentally conserved.

Despite the fundamental similarities in the numerical cognition of adults and young children, there is enormous conceptual change in children's numerical abilities from early childhood to adulthood [ 32, 33]. For example, by 3 y, children have memorized some portion of the count sequence from one to ten, yet they cannot verbally count to construct a set of items [ 40– 42]. In fact, many children do not appreciate the link between number words and non-symbolic quantities by 5 y [ 43]. Children between the ages of 3½ and 4½ y have typically mastered the verbal count sequence to ten but make mistakes in the counting sequence between ten and 20 and have not yet mastered the ordinal structure of the decade count words (twenty, thirty, etc.) [ 44]. Lastly, accuracy and reaction time on non-symbolic numerical tasks change dramatically between the ages of 2 and 7 y [ 32, 33, 45].

By adulthood, humans perform rapid, nonverbal numerical computations across a wide range of stimuli, sensory modalities, and numerical values with great precision [ 11]; they are also proficient at manipulating numerical symbols in complex mathematical operations. Thus, while certain aspects of numerical performance (such as ratio-dependent discrimination) remain constant over development, there is also a great deal of developmental change in numerical competence. Developmental changes in numerical competence may relate to changes in the brain regions involved in numerical processing regardless of whether symbolic and non-symbolic numerical stimuli are processed by a common substrate in adulthood. Therefore, an important question is to what extent a brain region known to be important for numerical approximation in adults, the IPS, shows continuity in function over development [ 17].

Little is known about how the child's brain comes to perform the complex mathematical feats of human adults, with only one study investigating the neural correlates of numerical processing in pre-school children [ 14]. That study employed scalp-recorded event-related potentials. Event-related potentials provide exquisite information about the timing of mental processes but lack the spatial resolution for determining the precise locus of number-related activity in the brain. Localizing numerical processing to specific brain structures using techniques such as function magnetic resonance imaging (fMRI) is crucial for determining whether common neural circuits are responsible for numerical performance both early in development and in adulthood. While there have been several fMRI studies of numerical processing in adult humans implicating the IPS as a basis of fundamental numerical processing, there has never been a parallel fMRI study of numerical processing in pre-school children. Such a study is essential for addressing the question of whether number-related activity in the IPS is a *source* of fundamental numerical abilities or is, instead, a *consequence* of the more sophisticated numerical abilities exhibited in adulthood.

**Figure 1:**
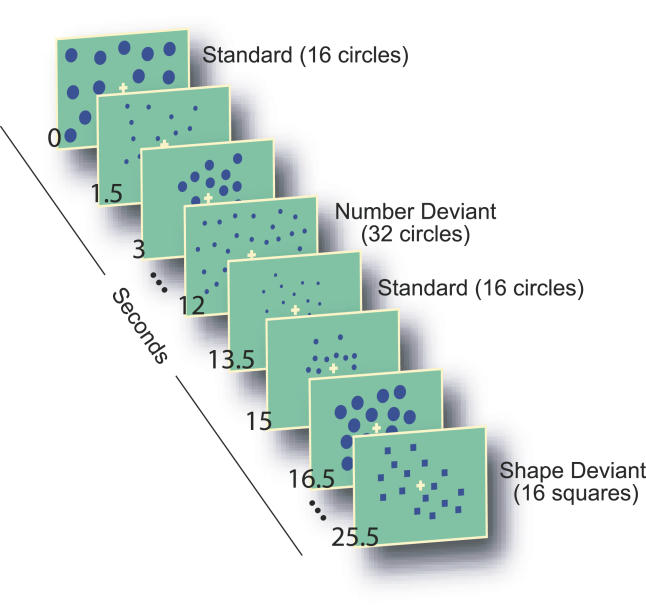
Task Design Participants were given the experiment-irrelevant task of fixating on a central crosshair and pressing a joystick button when the crosshair turned red. They passively viewed a stream of visual arrays, the majority of which contained the same number of elements and element shape. Occasionally, a stimulus was presented that deviated from the standard stimuli in *either* number of elements (number deviants) *or* local element shape (shape deviants). Cumulative surface area, density, element size, and spatial arrangement varied among standard stimuli so that participants were not habituated to these dimensions. Deviant and standard stimuli overlapped in cumulative surface area, density, element size, or spatial arrangement so that these dimensions were never novel for deviant stimuli. Number deviants differed by a 2:1 ratio from the standard number of elements such that half of the numerical deviants had a greater number of elements than the standard, and the other half had fewer elements. Elements in standard arrays were circles while shape deviants contained squares or triangles.

In this study, we investigated whether the IPS responds to non-symbolic numerical value in number-sophisticated adults and 4-y-old children who have limited experience using symbolic numbers. We sought to determine whether the IPS responds to numerical values (1) when presented non-symbolically as visual sets of elements and (2) before sophisticated symbolic and non-symbolic numerical abilities emerge. We used an event-related fMRI adaptation paradigm at 4-Tesla to measure differences in the IPS response to stimuli that were novel in number compared to stimuli that were novel in shape. Children and adults were tested with identical imaging paradigms. Children ( *n* = 8) and adults ( *n* = 12) passively viewed a constant stream of visual element arrays ( Figure 1). Arrays consisted of blue circle elements that varied in density, cumulative surface area, spatial arrangement, and size, but were constant in both the number of elements (16 or 32) and in local element shape (circles). Thus, participants adapted to the constant number and shape of the elements.

Occasionally, a deviant stimulus was presented that differed from the standard stimuli either in the number of elements (number deviant) or in the local element shape (shape deviant). Number deviants differed by a 2:1 ratio such that half of the number deviants had a greater number of elements than the standard stimuli whereas the remaining half had fewer elements. The standard elements were circles, whereas half of the shape deviants consisted of square elements, and the remaining half were triangles. Number and shape deviants were presented with equal frequency. The density, cumulative surface area, and element size of the standard stimuli were continuously varied (i.e., each dimension changed every 1.5 s) to prevent neural adaptation to these dimensions. The values of cumulative surface area, density, and element size for deviant stimuli overlapped with the values of these dimensions for the standard stimuli. The cumulative surface area of deviant stimuli was equated with the middle value of the standard stimuli and the values for the density and element size of the deviant stimuli were from the same distribution as the standard stimuli. Therefore, the only dimensions that repeated among the standard stimuli were the number and shape of the elements, whereas the only dimensions of the deviant stimuli that were novel, compared with standard stimuli, were the number or shape of the elements. Children and adults were asked to maintain fixation on a central cross hair. To ensure that they attended to the stimulus display, they were asked to press a button when the central cross hair turned red. We examined which brain areas responded exclusively to each class of deviant stimuli in adults and children. Then, we compared children's results with those of our adult participants. Further methodological detail is provided in Materials and Methods.

## Results

### Adults' fMRI Results

**Figure 2:**
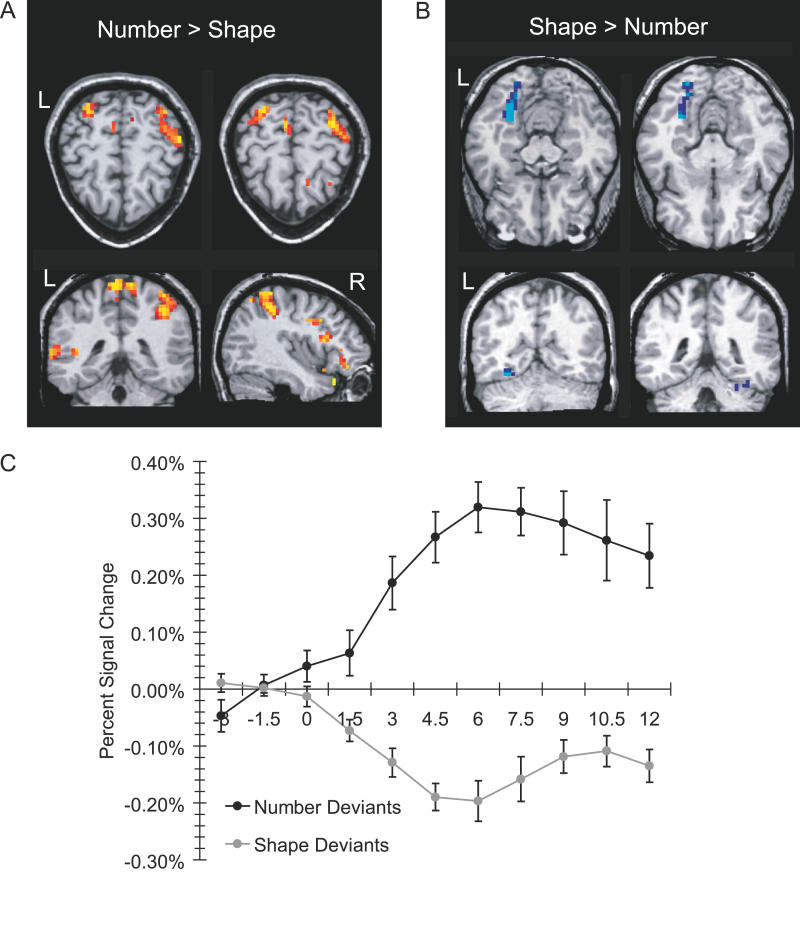
Adult Participant's fMRI Results (A) Regions that were more active during the presentation of number compared to shape deviants ( *p* < .05, cluster size > six functional voxels). (B) Regions that were more active during the presentation of shape compared to number deviants ( *p* < .05, cluster size > six functional voxels). (C) Time course of activity (percent signal change) for number-selective (number > shape) regions in the IPS, averaged from individually-drawn functional regions of interest from the IPS, from 3 s pre-stimulus to 12 s post-stimulus.

In adult participants, regions in and around the IPS (bilaterally) showed significantly greater activity to number deviants than to shape deviants. A random-effects analysis that directly compared activity to number and shape deviants revealed bilateral number-related activity localized to the IPS and extending into the inferior and superior parietal lobules consistent with previous studies that tested adults with Arabic numeral stimuli, symbolic arithmetic operations, and number words [ 21, 23], as well as a study of non-symbolic number processing [ 28] ( Figure 2A, MNI coordinates x, y, z: 43, −47, 59, BA [Broadman's area] = 40; −31, −66, 62, BA = 7). Also consistent with previous studies [ 28, 29], regions of activity that responded exclusively to shape deviants were localized to the ventral temporal-occipital cortex including the fusiform (32, −70, −14, BA = 19; −34, −47, −19, BA = 37) and lingual gyri (−27, −72, −7, BA = 18) ( Figure 2B).

Figure 2C shows the time course of blood oxygenation level-dependent (BOLD) response to number versus shape deviants in the IPS (defined on a participant-by-participant basis) for time points occurring up to 12 s post-stimulus. Between 3 and 7.5 s post-stimulus onset, the IPS produced a significantly greater response to stimuli in which the number of elements changed but the shape remained constant, than to stimuli in which the local element shape changed but the number of elements remained constant. This value was significantly greater than the baseline level of activity across participants (mean = .30%, *t* (11) = 6.63, *p* < .001) whereas the hemodynamic response (HDR) to shape deviants in this region was significantly lower than baseline (mean = −.17%, *t* (11) = −6.14, *p* < .001).

Shape deviants had the same number of elements as the standard stimuli. Thus, the decreased response to shape deviants in the IPS likely resulted from a decreasing response to repeated presentations (1 per 1.5 s) of a numerical value, relative to a baseline that was set to zero in the baseline-subtracted epoch averages (but may actually have been much greater than zero in this rapid presentation, event-related paradigm). This interpretation is consistent with the predictions of an fMRI adaptation design for decreased responding over time with increasing presentations of a stimulus [ 46, 47]. The adaptation of a BOLD signal increases gradually as the number of repetitions increases [ 46– 48]. Task- and stimulus-related decreases in BOLD contrast (deactivations) have been reported in previous fMRI studies [ 49– 54]. Deactivations have also been correlated with decreased blood oxygenation and neural suppression [ 54]. Therefore, the waveforms from the present study may reflect relatively high baseline activity that increases slightly when a numerical deviant is presented but decreases significantly with repeated presentations of the same numerical stimulus (i.e., continued adaptation), even in the presence of a non-numerical change (i.e., shape change). In any case, the IPS response to numerical deviants is significantly greater than baseline and significantly different from the IPS response to shape deviants. This result indicates that the IPS preferentially responds to non-symbolic numerical stimuli.

Activity to deviant stimuli was not asymmetrically influenced by one kind of number deviant or one kind of shape deviant. There were no significant differences in activity between the two kinds of number deviants ( *t* (11) = .95, *p* = .36) in the IPS or the two kinds of shape deviants ( *t* (11) = .44, *p* = .67) in the fusiform and lingual gyri. Thus, the IPS responded to deviations in number whether the deviant stimulus contained a larger or smaller number of elements. Similarly, the ventral temporal-occipital cortex responded to deviations in local element shape whether the deviant elements were squares or triangles.

In summary, the IPS of adult participants showed significantly greater activity to numerical deviants than shape deviants. As reviewed above, the IPS is known to respond selectively to symbolic numerical stimuli such as Arabic numerals and number words. The IPS response to non-symbolic numerical deviants in adult participants demonstrates that the IPS responds to numerical values independently of notation.

### Children's fMRI Results

**Figure 3:**
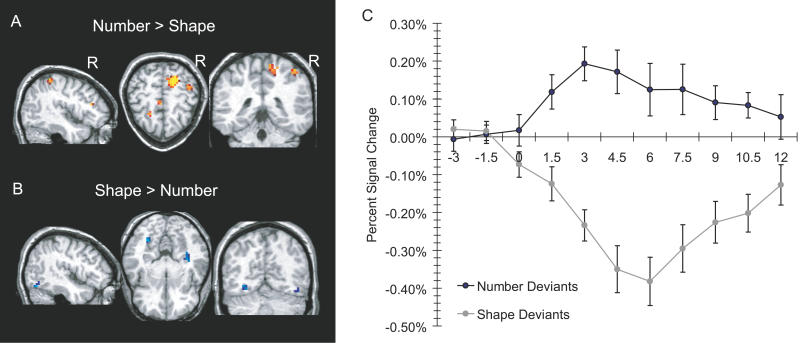
Child Participant's fMRI Results (A) Regions that were more active during the presentation of number compared to shape deviants ( *p* < .05, cluster size > six functional voxels). (B) Regions that were more active during the presentation of shape compared to number deviants ( *p* < .05, cluster size > six functional voxels). (C) Time course of activity (percent signal change) for number-selective (number > shape) regions in the IPS, averaged from individually-drawn functional regions of interest from the IPS, from 3 s pre-stimulus to 12 s post-stimulus.

**Figure 4:**
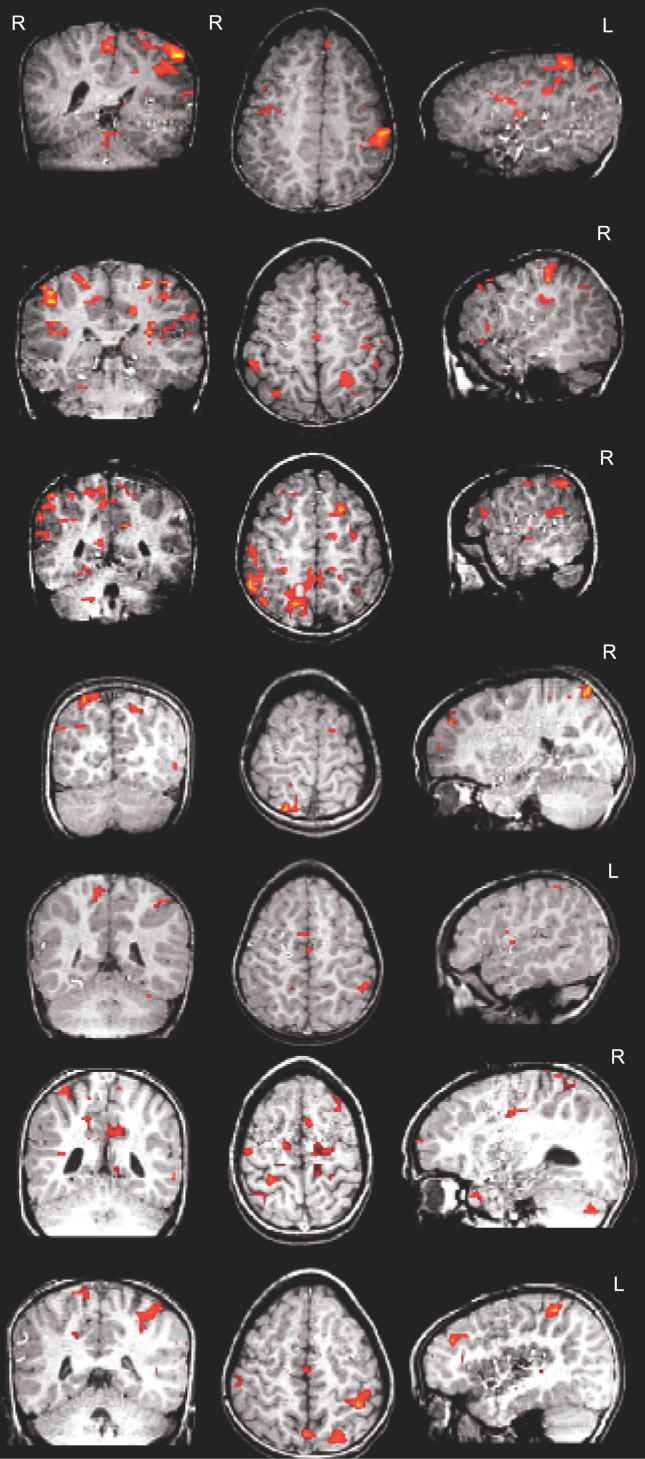
Data from Individual Children Each row presents an individual participant's activation map indicating regions that were more active during the presentation of number compared to shape deviants ( *p* < .05, cluster size > eight functional voxels) overlaid on that child's own anatomical images. One child moved during the anatomical scan (which occurred after the functional scan) and is thus not included in this figure.

We performed direct, random-effects contrasts between number and shape deviants for child participants as described above for adults. The average activations across participants are presented in Figure 3A and 3B in a common adult template brain space. This analysis revealed significant activity evoked by numerical deviants ( Figure 3A; number > shape) in and around the right IPS (MNI coordinates: 45, −44, 62, BA = 5) and right superior parietal lobule (SPL) (18, −53, 65, BA = 7). We also found significant activations to numerical deviants in the left precentral gyrus (−56, 2, 62, BA = 6), left superior frontal gyrus (−21, 1, 62, BA = 6), left medial frontal gyrus (0, −14, 57, BA = 6), left inferior parietal lobule (−62, −28, 25, BA = 40), and right middle frontal gyrus (46, 28, 20, BA = 46); although we had no a priori hypotheses regarding the roles of these latter regions in numerical processing. Activation maps that reveal significant number-related activity for individual children overlaid upon their own anatomical images (without spatial normalization) are presented in Figure 4. As illustrated, each child exhibited significant number-specific activity in and around the IPS.

Children showed significantly greater activity to changes in the local shape of the elements compared with changes in numerical value (shown in Figure 3B) in the left lateral occipital-temporal complex (−39, −78, 4, BA = 19 and −41,−88, 13, BA = 18) and right fusiform gyrus (45, −71, −11, BA = 19). In addition, we identified significant shape-specific activations in the right anterior cingulate (17, 39, 11, BA = 32), right superior frontal gyrus (15, 28, 62, BA = 6), and right caudate (37, −38, −1). Thus, in 4-y-old children, shape adaptation effects appear to occur in similar regions as those reported in adults (2,46).

Figure 3C shows the time course of number deviant and shape deviant activity in the IPS. As compared to adults, children showed a similar BOLD response to deviants in the IPS: the response to number deviants was significantly greater than baseline (mean = .15%, *t* (7) = 3.62, *p* < .01) while the response to shape deviants was significantly below baseline (mean = −.30%, *t* (7) = −7.73, *p* < .001). Thus, the IPS continued to habituate to the constant numerical value of the shape deviant stimuli but showed an increased response to the novel numerical value of the number deviant stimuli.

A comparison of activity between the two kinds of numerical deviants revealed no difference in the IPS ( *t* (7) = .24; *p* > .81) or SPL ( *t* (7) = −.89; *p* > .41) between the larger and smaller numerical deviants. Thus, the IPS responded to numerical deviants of a 2:1 ratio regardless of the absolute magnitude of the numerical values. Similarly, a comparison of activity between the two kinds of shape deviants revealed no significant difference in the lateral occipital complex ( *t* (7) = .38; *p* > .72) or fusiform gyrus ( *t* (7) = .75; *p* > .48). These analyses indicate that numerical activity was not related to an increase in visual attention to the number of array elements nor was shape activity specific to a particular shape. Instead, children's number- and shape-related activity in these regions encompassed the broad classes of deviants in our study.

To summarize, children showed greater activation in the IPS to numerical deviants than to shape deviants. Number deviant activity was significantly greater than baseline and significantly differed from activity related to shape deviants in the IPS. Our results show that by the age of 4 y, children show selective activation of the IPS in response to non-symbolic numerical values.

### Comparison of IPS Activity in Children and Adults

**Figure 5:**
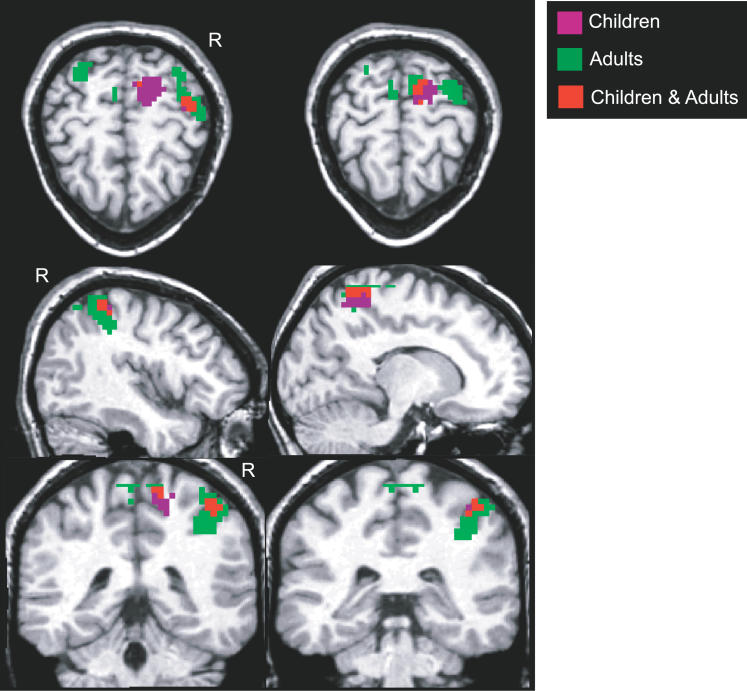
Child and Adult Number-Selective Brain Regions (Number > shape from Figures 2 and 3) plotted in same space. Adults showed more extensive areas of activation than children; however, the same brain regions were active for children as for adults in this study.

Children's number-related activity in the IPS was strikingly similar to activity in adult participants tested under identical conditions. Figure 5 shows brain regions activated by numerical deviants for children and adult participants in the present study, tested with identical tasks and stimuli. While adults tended to show more extensive activations, numerical activity in the IPS and SPL overlapped considerably in children and adults. Within the right IPS region, adults and children overlapped for their number > shape responses on a total of 112 voxels. In the right IPS, where children overlapped considerably with adults, the extent of the activation was greater for adults, with adults activating approximately five times as many voxels as children (586 versus 112 voxels). Note, however, that the more extensive activation for adults is possibly due to the larger sample of adult participants (12 adults versus eight children). The key finding here is that the IPS activity in children exhibited substantial overlap with that of adults.

The MNI coordinates for the IPS activations in adults were 43, −47, 59 and −31, −66, 62; which can be compared to the coordinates of the children's IPS (45, −44, 62) and SPL (18, −53, 65) activations. The locus of number-related activity in our pre-school participants was also comparable to adult activity reported in similar studies of non-symbolic numerical processing (1: 36,−60, 52; 28, −56, 44; 16, −56, 44) and to activity related to basic mathematical ability in adults (22: 44, −36, 52; 20, −60, 60; −56, −44, 52). This finding suggests that the neural circuitry for processing non-symbolic numerical information is organized similarly to adults by at least 4 y.

One noteworthy difference between the number-related brain activity of children and adults in our study is that adults showed robust bilateral activation in the IPS while children, on average, showed number-related IPS activation predominantly in the right hemisphere. To evaluate the statistical significance of this group difference in the laterality of activation patterns, we conducted a between-groups random-effects analysis comparing the levels of activity at peak magnitude in response to numerical deviants for children and adults. This analysis confirmed the impressions given by Figure 5: adults had significantly more activity bilaterally in the IPS region and children had less activity, on average, in the left IPS region and more bilateral activity in the SPL region. The MNI coordinates for regions of adults > children and children > adults number-related activity are provided in Table 1.

**Table 1:**
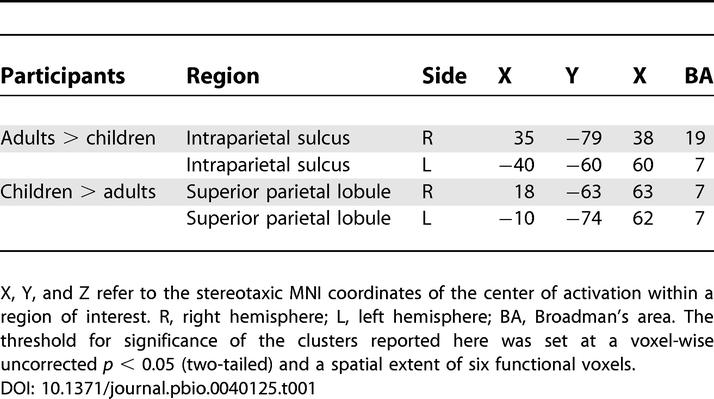
Summary of a Random Effects Analyses Contrasting Number-Related Activity in Parietal Regions for Children and Adults

One recent study also found hemispheric asymmetries in the IPS associated with 8- to 19-y-old children's developing numerical abilities [ 55]. Rivera and colleagues (2005) demonstrated that an inferior parietal region including the left IPS (54: −51, −37, 46) becomes increasingly responsive during symbolic mathematical operations between 8 and 19 y, whereas a corresponding region in the right hemisphere is equally active during mathematical processing at all ages. This study suggests that the left hemisphere may become functionally specialized for mathematical processing over development while the right hemisphere shows little developmental change. Given our finding that more 4-y-old children show right IPS activation while adults show bilateral IPS activation, one possibility is that non-symbolic numerical processing follows a similar trajectory of hemispheric specialization over development. However, some studies have found that the right IPS is also more active than the left IPS during numerical processing in adults [ 56]. Further, as shown in Figure 4, some children exhibited more number-related activity in the left IPS than others. Therefore, this aspect of our results should be viewed cautiously until subsequent studies can confirm that the right lateralization of number-related IPS activity is unique to young children.

Additionally, as described further in Materials and Methods, we spatially normalized the data from children and adults into a common adult template to perform a direct comparison between these two groups. Although the practice of normalizing fMRI data from children to make this sort of direct comparison has been validated by a few previous studies in older children [ 57, 58], we cannot rule out the possibility that the nonlinear warping procedures used in the present study slightly shifted relevant brain loci between the children and adults. If this were true, the observed differences between adults and children might be explained, in part, by the spatial normalization procedures. This possibility seems unlikely, however, given the relatively coarse resolution of the fMRI data relative to the anatomical differences caused by spatial normalization reported in prior studies [ 57, 58]. If the differences between children and adults resulted from spatial normalization, there actually may be more overlap in the parietal activation of children and adults because adults showed greater number-related activity in the IPS than children, while children showed greater number-related activity than adults at an adjacent SPL site. Thus, although the between-groups random effects analysis revealed differences in the amount of activation at IPS and SPL sites for children and adults, our main finding is that the topographical pattern of parietal activation is remarkably similar between children and adults.

### Behavioral Testing of Children

We tested the same children who participated in the fMRI study on a non-symbolic numerical discrimination task using the same numerical values from the fMRI session (8, 16, 32, and 64). Children were presented with two arrays of dots on a touch-screen monitor and were instructed to choose the array with the larger number of dots on each of 50 trials. Density, cumulative surface area, cumulative perimeter, and element size were carefully controlled. Overall, children performed significantly above chance (chance = 50%; mean = 89%; *t* (7) = 6.89; *p* < .001), although one child scored near chance. This result is consistent with previous studies demonstrating non-symbolic numerical proficiency in young children [ 31– 33, 43, 45, 59].

We also investigated children's knowledge of the verbal counting sequence. Verbal counting ability was assessed for each child using the “How high?” and “How many?” tasks of Wynn [ 40]. In the “How high?” task, children were asked to recite the verbal counting sequence from memory beginning with “One” whereas in the “How many?” task, children were asked to verbally count a set of 30 items. For both tasks, we recorded the highest number to which children could count without making an error. On the “How many?” task, three children successfully counted all 30 items without making an error (i.e., counting an item or count word twice or skipping an item or count word) in the counting sequence; one child successfully counted to 14; two children counted to 11; and the remaining two children counted to ten without making an error. On the “How high?” task, two children counted to 100 before being stopped by the experimenter; two children counted to 30 without making an error; one child to 20; and three children counted to less than 15 before making an error.

Overall, our behavioral tests revealed robust nonverbal numerical competence among children for the target numerical values ranging from eight to 64. However, the majority of children in this study could not count verbally to 64. The numerical values tested in the fMRI session were thus outside the range of verbal counting proficiency for many of these children, yet children showed a number-selective response in the IPS to these values when presented as numerical deviants.

## Discussion

A critical question for the study of numerical cognition is whether the complex, symbolic mathematical abilities of adult humans share a neurobiological and developmental origin with non-symbolic numerical abilities. A growing body of evidence suggests that the ability to judge numerical values nonverbally was an important evolutionary precursor to adult human symbolic numerical abilities [ 15, 60, 61], and that it is a language-independent cognitive capacity [ 7, 9]. Our study provides additional evidence that there is an important neurobiological link between symbolic and non-symbolic numerical cognition in adults and thus helps to resolve current controversies in the adult literature [ 28, 29]. Most importantly, our study further demonstrates that the IPS is recruited for non-symbolic numerical processing early in development, before formal schooling has begun.

A great many studies have investigated how children acquire the verbal counting system [ 2, 36, 44]. Furthermore, there are disparate hypotheses about how children begin to map the meaning of number words onto nonverbal representations of number [ 4, 35, 36, 44, 62]. Our study provides new evidence of a neurobiological link between the early approximate numerical abilities of children, and the more sophisticated non-symbolic and approximate symbolic numerical abilities of adults. We therefore suggest that non-symbolic numerical activity in the IPS may be a developmental origin of adult mathematical knowledge [ 15, 17]. Ultimately, a full description of how children learn the meaning of number words must incorporate these new findings.

As mentioned, previous work with adult participants has implicated the IPS in processing Arabic numerals and number words but has remained inconclusive on the role of the IPS in processing non-symbolic number [ 28, 29, 30]. One study [ 28] used an event-related adaptation design and found that activity in the IPS correlated with the numerical distance between standard numerical stimuli and numerical deviants. However, unlike the present study, in the study by Piazza and colleagues [ 28], number-related activity in the IPS was not directly contrasted with activity related to a class of non-numerical stimuli in a random-effects analysis and compared against baseline activity. Thus, the authors could not definitively demonstrate number-specific brain activity for visual arrays. Similarly, number specificity in the IPS was not definitively demonstrated in a study by Ansari et al. [ 30], which nicely demonstrated parametric modulation of IPS activity by numerical distance but did not test whether the IPS responds significantly above baseline to non-numerical stimuli. The current study directly contrasted number-related activity in the IPS with activity related to shape changes in a random-effects analysis and compared this against baseline activity, providing strong evidence in favor of the argument that the IPS is sensitive to numerical changes for sets of visual objects and that this region does not respond equally to all stimulus changes (i.e., shape changes; [ 28]).

In contrast to our result, Shuman and Kanwisher [ 29] found no difference in IPS activity between blocks in which number varied and those in which number was held constant. One possible explanation of the conflicting results between these two studies is that in the study by Shuman and Kanwisher [ 29], surface area varied in the “number constant” blocks, which could have elicited IPS activity because the IPS has been shown to respond to changes in surface area [ 63]. Consequently, the IPS may have responded to number changes in the “number varied” condition, but responded to surface area changes in the “number constant” condition. These two responses in the IPS could have canceled each other out in a statistical contrast [ 30]. This explanation of the Shuman and Kanwisher [ 29] result assumes that the IPS plays a more general role in magnitude judgments and is not selective for number per se. Yet it leaves open the possibility that the IPS responds to changes in magnitude as suggested by Pinel and colleagues [ 63] but not to changes along other dimensions (e.g., shape).

Alternative explanations of the IPS response to numerical deviants such as a non-numerical recovery response, a non-numerical novelty response, a general novelty response, or a response reflecting changes in visual attention cannot account for our result. First, the recovery response exhibited by the IPS to numerical deviants cannot be attributed to non-numerical dimensions such as cumulative surface area, density, or element size because these non-numerical dimensions were constantly varied in the standard stimuli while number and shape were held constant. Thus, given the known characteristics of an fMRI-adaptation design [ 48], the IPS could not adapt to these dimensions. Second, the IPS response to numerical deviants cannot be explained as a novelty effect evoked by the non-numerical dimensions of cumulative surface area or density because shape and number deviants were equated on these dimensions and their effect, if any, would cancel out in the number > shape contrast. The element size of the deviant stimuli would also fail to evoke a novelty response because element sizes for deviant stimuli were taken from the distribution of values from the standard stimuli and were thus never novel compared to the range of standard stimuli to which the IPS adapted. Third, because we directly contrasted the brain response to two categories of deviant stimuli, alternate explanations of IPS activity, such as a general novelty effect, cannot easily account for our result. Additionally, the IPS responded to numerical deviants regardless of whether deviants increased or decreased in their number of elements from the standard stimuli. This result indicates that a greater IPS response to numerical deviants does not simply reflect increased attention with the number of visual objects presented. Lastly, our study did not require participants to perform an explicit number-related task, indicating that task difficulty is not necessarily a correlate of IPS activity. An implication of this finding is that the IPS responds automatically to numerical information when participants passively view numerically relevant stimuli. Taken together with previous studies, our results suggest that the IPS plays a role in both symbolic and non-symbolic numerical processing and is thus important for processing number independent of notation. Further, our comparable results from both children and adults make a case for an early-developing neural substrate for notation-independent numerical processing.

Behavioral studies of children's developing numerical abilities have highlighted important similarities and differences in numerical competence over development [ 4, 17, 35, 59]. Studies of numerical processing in adults have revealed number-selective brain regions in and around the IPS [ 28, 21, 30]. Our study provides evidence that numerical processing invokes a common neural substrate in adults and children during the presentation of non-symbolic numerical stimuli; by 4 y, the IPS responds more strongly to numerical changes than to shape changes. Therefore, the similarities in numerical performance across ontogeny may reflect reliance on a single substrate for numerical processing from childhood to adulthood. However, symbolic numerical abilities are reported to recruit a broader network of number-specific brain regions than the IPS alone [ 21]. For example, the ability to solve multiplication tables and other math facts appears to recruit regions in and around the left angular gyrus [ 20, 21, 64– 66]. Some researchers have suggested that this brain region is important for the explicit manipulation of numerical values that is characteristic of adult human mathematics [ 35, 66]. Thus, conceptual development related to cultural, linguistic, and symbolic numerical practices might cause changes in the network of brain regions involved in precise, sophisticated adult mathematics [ 2, 20]. However, the neural basis of notation-independent numerical processes in the IPS may be the nucleus of this sophisticated mathematical network over development.

It is possible that the IPS also supports non-symbolic numerical discrimination in infancy. As mentioned, the behavioral signatures of non-symbolic numerical processing in infants, children, and adults indicate that numerical discrimination employs similar psychological mechanisms over development. Studies with human infants in the 6th mo of life have demonstrated that, like adults and children, infants also show ratio-dependent numerical discrimination [ 37– 39]. Similarly, studies of non-human animals also show striking parallels in the behavioral and neural signatures of number processing [1–8,15,16,67–69]. The neural bases of numerical cognition may be, therefore, both ontogenetically and phylogenetically primitive.

In conclusion, our data provide strong evidence in favor of the view that the IPS, known to be part of a cerebral network important for symbolic number processing, is also recruited in non-symbolic numerical processing. Further, by testing one of the youngest samples of healthy children in a cognitive fMRI study, we have shown that by 4 y, the IPS is already recruited when children represent number non-symbolically. Our results are therefore consistent with the view that the IPS is the ontogenetic and phylogenetic origin of non-symbolic number processing and serves as a foundation upon which symbolic number processing is built. Although our data further demonstrate the ubiquitous role of the IPS in numerical processing, additional work is necessary to determine whether any region of the IPS is truly number-specific or instead plays a more general role in magnitude processing.

## Materials and Methods

### Participants

Twelve healthy young adult volunteers (five females, seven males; *M* = 25 y, range = 21–37 y) and eight typically developing 4-y-old children (five females, three males; *M* = 4.75 y, range 4.25–4.95 y) participated in this study. All participants had normal or corrected-to-normal vision and were screened against neurological and psychiatric illnesses. The parents of the child participants gave informed consent prior to participation, and the families were given a toy as a token of our appreciation and financial compensation for their time. Adult volunteers gave informed consent and were given financial compensation for their time. The Institutional Review Board of Duke University Medical Center, Durham, North Carolina, United States approved this project.

In all, 17 children were brought in for scans. They were first trained in our mock scanner. The practice scans are described in more detail below. Of these 17 children, only anatomical scans were obtained for two children. All 15 of the remaining children performed both functional runs, although seven of them moved excessively during both runs and could not be used in the analyses. All 12 adults provided useable data. Comparison of movement values for the adults and children revealed no significant differences in the amount of movement between these two groups during the scans.

### Experimental design

Stimuli were visual arrays of circles ( Figure 1). The experiment consisted of two blocks counterbalanced for the numerical quantity of the habituation arrays (16 or 32). Each block consisted of 238 stimuli presented at a rate of 1 every 1,200 ms for a duration of 300 ms. Participants fixated on a central fixation cross and were given the experiment-irrelevant task of pressing a joystick button when the central fixation cross turned red to ensure that they attended to the stimuli. This happened three times per block: once near the beginning of the block, once in the middle, and once near the end of the block.

Twenty deviants were presented in each block. Half of the deviants differed from the habituation stimuli in their number of elements. The other half differed in the local shape of the elements. Deviant stimuli occurred randomly in the stimulus train with the constraint that two successive deviants were separated by at least eight and at most 11 habituation stimuli. Deviants appeared in a pseudo-random order with each type of deviant presented once without replacement and then the deviant order was re-randomized. The numerical quantity of the elements in number-deviant stimuli differed from the habituation quantity by a ratio of 2:1. Thus, for blocks in which the number of elements in the habituation stimulus was 16, half of the number deviants contained eight elements and the other half contained 32 elements, while for blocks with a habituation number of 32, half of the number deviants contained 16 and the other half contained 64 elements. The local element shape of habituation stimuli was circles. Half of the shape deviants had square elements and the other half had triangular elements. All deviant stimuli were equally probable.

Segments of the IPS have been shown to respond to changes in continuous magnitude such as surface area, in addition to the numerical magnitude represented by Arabic numerals [ 63]. To ensure that participants were being habituated to numerical magnitude and not non-numerical magnitude of arrays, we varied the cumulative surface area, element size, and density of the elements within each array across habituation trials. For habituation arrays, there were seven different values for element size and cumulative surface area and three different values for density (Standard stimuli: cumulative area, range = 15,000–60,000 pixels; element size, range = 937.5–3,750 pixels; density, range = 0.00007–0.00025 pixels). The different values for these dimensions were presented pseudo-randomly in that they were randomly ordered and presented without replacement, and then re-randomized. The values of these dimensions for all deviant stimuli overlapped with the range of values for habituation stimuli (Deviant stimuli: cumulative area = 30,000 pixels; element size, range = 937.5–3,750 pixels; density range = 0.00007–0.00025 pixels). All deviant stimuli were equal in cumulative surface area (30,000 pixels) and the value chosen was equal to the middle value used for habituation stimuli. For example, a pseudo-randomly ordered sequence of eight standard stimuli with a constant number (16) and local element shape (circles) could have the values (in pixels) 2,727.5, 1,250, 937.5, 2,250, 1,875, 3,750, 1,500, and 2,727.5 for element size; 43,640, 20,000, 15,000, 36,000, 30,000, 60,000, 24,000, and 43,640 for cumulative surface area; and .00025, 0.0001, 0.00007, 0.0001, 0.00025, 0.00007, 0.00025, and 0.0001 for density. A number deviant stimulus with eight circles following such an array would have an element size of 3,750, a cumulative surface area of 30,000, and a density of 0.00007 in pixels. Thus, the only candidate dimension for neural adaptation in the standard stimuli was the number and shape of the elements; similarly, the only novel dimension of the deviant stimuli was the number or shape of elements.

### Imaging protocol

Scanning was performed on a General Electric Health Technologies, 4T LX NVi MRI scanner system, equipped with a quadrature birdcage radio frequency head coil. Sixty-eight high-resolution images were acquired using a 3D fast SPGR pulse sequence (TR = 500 ms; TE = 20 ms; FOV = 24 cm; image matrix = 256 ^2^; voxel size = 0.9375 × 0.9375 × 1.9 mm). Whole brain functional images were acquired using a gradient-recalled inward spiral pulse sequence [ 70, 71] sensitive to BOLD contrast (TR, 1,500 ms; TE, 35 ms; FOV, 24 cm; image matrix, 64 ^2^; α = 62 °; voxel size, 3.75 × 3.75 × 3.8 mm; 34 axial slices). These functional images were aligned to the structural images.

### Preparing children for fMRI scans

Acquisition of neuroimaging data from children involves several methodological challenges. Perhaps the most noteworthy of these is the child's compliance with the requirement to remain motionless during the scan. A key methodological advance in our laboratory's establishment of child neuroimaging research has been to develop “mock scanning” facilities. We constructed an MRI simulator for use in acclimating children to the scanner environment and for training these participants to minimize head motion. We also developed a protocol and computer software for use with the MRI simulator to limit head motion by training children to remain still during fMRI scanning. Children were “trained” using operant-conditioning procedures implemented in custom-written software that receives input from a head motion sensor and uses that input to direct the operation of a video player. The child watched a favorite movie, and the movie was halted whenever the child exhibits head motion above a progressively stricter threshold.

### Data analysis

Image preprocessing was performed with custom programs and SPM 99 modules (Wellcome Department of Cognitive Neurology, United Kingdom). Images were time-adjusted to compensate for the interleaved slice acquisition and realigned to the tenth image to correct for head movements between scans. The realigned scans were then spatially normalized to the Montréal Neurologic Institute (MNI) template found in SPM 99 using the standard two-part procedure involving first a 12-parameter affine registration for global normalization followed by a non-linear basis function registration for regional transformations. The functional data were high-pass filtered and spatially smoothed with an 8 mm isotropic Gaussian kernel prior to statistical analysis. Except where otherwise noted, these normalized and smoothed data were used in most of the analysis procedures described below. By normalizing the children's imaging data to the MNI space, we were able to compare functional activation foci in children and adults within a common template. Kang et al. [ 57] recently provided an empirical validation of normalization for analysis of fMRI data from children. Kang et al. found very small differences (relative to the resolution of fMRI data) in the spatial correspondence among several brain loci between young children and adults after a standard, nonlinear transformation that warped child and adult fMRI data into a common adult Talairach space. Based on these and other similar findings [ 56], we directly compared data from adults and children in common, adult stereotactic space in this study.

The primary analysis consisted of a random-effects assessment of the differences between the shape and number deviant conditions at the expected peak of the hemodynamic response (HDR). This analysis consisted of the following steps: (1) The epoch of image volumes beginning 3.0 s before and 12 s after the onset of each deviant stimulus was excised from the continuous time series of volumes. (2) The average intensity of the HDR at expected peak was computed for the time interval ranging from 4.5–7 s by deviant type. A *t*-statistic was then computed at each voxel within the brain to quantify the HDR differences between shape and number deviants. This process was performed separately for each participant. (3) The shape > number and number > shape *t*-maps were then subjected to a random-effects analysis that assessed the significance of differences across participants. To reduce the number of statistical comparisons and thus the false-positive rate, the results of the random-effects analyses were then restricted to only those voxels in which a significant ( *p* < .05, uncorrected) HDR was evoked by either of the two conditions. The threshold for significance of a difference in the HDR peak was set at *p* < .05 (two-tailed, uncorrected) and a minimal spatial extent of six uninterpolated voxels. We performed this analysis separately for the adult and child samples. We localized each cluster of number > shape and shape > number activation by anatomical location, MNI coordinates of the center of the activation, and BA.

We also conducted a between-participant random-effects analysis to evaluate the statistical significance of observed differences between adults and children in patterns of number-related activity in the parietal cortex. This analysis compared levels of number-related activity at the peak of the HDR (4.5 s −7 s). For this analysis, we randomly selected eight of our 12 adult participants, so that the samples sizes would be equivalent for adults and children. The threshold for significance of adult versus child (adults > children, children > adults) difference in the HDR peak was set at *p* < .05 (two-tailed, uncorrected) and a minimal spatial extent of six voxels.

For an additional set of analyses, we used the acquisition aligned and motion-corrected, un-normalized imaging data. Using this data, overlaid on each participant's own anatomical images, we identified, on a participant-by-participant basis, regions of activation within the IPS that were: (1) significantly above baseline in their response to number ( *p* < .05, uncorrected), (2) exhibited significantly greater activity to number compared to shape deviants at expected peak ( *t* = 1.96, *p* < .05, uncorrected), and (3) encompassed an area greater than eight functional voxels. The average shape and number epochs were then calculated for the voxels that meet these criteria and were averaged across participants for inspection.

## Abbreviations

BA: Broadman's area
BOLD: blood oxygenation level-dependent
fMRI: functional magnetic resonance imaging
HDR: hemodynamic response
IPS: intraparietal sulcus
MNI: Montreal Neurological Institute
SPL: superior parietal lobule
